# Non-infectious Intraocular Inflammation Following Intravitreal Anti-Vascular Endothelial Growth Factor Injection

**DOI:** 10.4274/tjo.galenos.2020.84042

**Published:** 2021-02-25

**Authors:** Mahmut Kaya, Ferit Hakan Öner, Betül Akbulut Yağcı, Ferdane Ataş, Taylan Öztürk

**Affiliations:** 1 Dokuz Eylül University Faculty of Medicine, Department of Ophthalmology, İzmir, Turkey

**Keywords:** Anti-VEGF, intraocular inflammation, topical steroid, vitritis

## Abstract

**Objectives:**

To evaluate the functional and anatomical results of patients with non-infectious intraocular inflammation (IOI) following intravitreal anti-vascular endothelial growth factor (anti-VEGF) injection for the treatment of neovascular age-related macular degeneration (nAMD).

**Materials and Methods:**

The medical records of patients receiving anti-VEGF treatment for nAMD between January 2015 and March 2019 were retrospectively analyzed. Preoperative and postoperative routine ophthalmological examinations, central macular thickness, duration of inflammation, and follow-up time of the patients with non-infectious IOI following anti-VEGF injection were recorded.

**Results:**

Non-infectious IOI was determined in 13 eyes (11 eyes with aflibercept, 2 eyes with ranibizumab) of 1,966 patients who received a total of 12,652 anti-VEGF (4,796 aflibercept and 7,856 ranibizumab) injections. IOI was detected after a mean of 7 injections (2-12 injections). All eyes had both anterior chamber reaction (Tyndall +1/+3) and vitritis (grade 1-3). None of the patients had pain, hypopyon, or fibrin reaction. Visual acuity progressed to baseline levels within 28.3 days. Vitritis continued with a mean of 40 days. All patients recovered with topical steroid therapy. In 11 eyes, injection of the same anti-VEGF agent was continued. No recurrence of IOI was observed in any patients.

**Conclusion:**

Non-infectious IOI following intravitreal anti-VEGF injection typically occurs without pain, conjunctival injection, hypopyon, or fibrin and responds well to topical steroid therapy. Visual acuity returns to baseline levels within weeks according to the severity of inflammation.

## Introduction

Non-infectious intraocular inflammation (IOI) is an acute, sterile inflammation that is not associated with an infectious agent and resolves without intravitreal antibiotic therapy. It occurs as a rare complication of intravitreal pharmacological agents.^1^ As more intravitreal injections are performed, the number of such side effects is also increasing.^1,2^ Non-infectious IOI has been reported in response to all anti-vascular endothelial growth factor (anti-VEGF) drugs ^1,3,4,5,6,7,8,9,10,11^, triamcinolone ^12,13^, and ocriplasmin.^14^ It typically occurs within a few days after injection, manifesting with visual impairment and vitritis without conjunctival injection and substantial pain.^3^ The reported incidence ranges from 0.09% to 0.55%.^1,8,9,10,11^

The aim of this study was to evaluate the frequency, characteristics, treatment, and functional and anatomical outcomes of non-infectious IOI after intravitreal anti-VEGF injection in patients with wet age-related macular degeneration (AMD).

## Materials and Methods

The medical records of 1966 patients who received intravitreal anti-VEGF (ranibizumab or aflibercept) injection for wet AMD in the retina unit of Dokuz Eylül University Department of Ophthalmology between January 2015 and March 2019 were retrospectively reviewed. The patients received a total of 12652 doses of anti-VEGF, and non-infectious IOI was detected in 13 eyes.

The study was conducted in accordance with the ethical standards stated in the Declaration of Helsinki after obtaining approval from the Dokuz Eylül University Ethics Committee. Patients aged 50 years and older who had wet AMD, were under ongoing anti-VEGF therapy and follow-up in our clinic, and were followed for at least 6 months were included in the study. Patients who did not receive the full anti-VEGF loading dose and those who had diabetes mellitus, uveitis, or history of ocular surgery in the last 6 months (cataract, glaucoma surgery) were excluded.

All anti-VEGF injections were performed under topical anesthesia with 0.5% proparacaine hydrochloride (Alcaine^®^, Alcon Laboratories Inc, Fort Worth, TX, USA) in the operating room under sterile conditions. Before the procedure, mydriasis was induced using 2.5% phenylephrine hydrochloride (Mydfrin^®^, Alcon Laboratories Inc, Fort Worth, TX, USA), 0.5% tropicamide (Tropamid^®^, Bilim Pharmaceuticals, Istanbul, Turkey), and 1% cyclopentolate hydrochloride (Sikloplejin^®^, Abdi İbrahim Pharmaceuticals, İstanbul, Turkey). Before injection, 5% povidone iodine was instilled in the lower fornix and left for at least 5 minutes. After cleaning the periocular skin and eyelids with 10% povidone iodine solution, a sterile eye drape was placed over the area and the eyelids were retracted using a blepharostat. Using a 30-gauge (G) needle, 0.05 ml of 0.5 mg ranibizumab or 2 mg aflibercept was administered in the superotemporal quadrant at a distance of 4.0 mm from the limbus in phakic eyes and 3.5 mm from the limbus in pseudophakic eyes. After administering the intravitreal injection, pressure was applied to the injection site with a sterile cotton-tip applicator while withdrawing the needle to prevent drug reflux and vitreous prolapse. Following injection, 5% povidone iodine was applied to the ocular surface. Patients were prescribed 0.3% ofloxacin (Exocin^®^, Allergan Pharmaceuticals Inc, Dublin, Ireland) eye drops and 1% fusidic acid (Fucithalmic^®^, Abdi İbrahim Pharmaceuticals, Istanbul, Turkey) viscous eye drops 6 times a day for 5 days. They were advised to present to our clinic immediately if they experienced vision loss, hyperemia, light sensitivity, or severe pain after the injection. Evaluations were performed at post-injection day 1, day 15, and day 30.

From the records of patients who developed non-infectious IOI after intravitreal anti-VEGF injection, the following data were recorded in detail: best corrected visual acuity (BCVA) measured during the inflammation; presence of conjunctival hyperemia and cells, lens status, and presence of cells in the anterior vitreous on slit-lamp examination; and the presence of vitritis, optic disc and macula findings, peripheral retinal findings, and vascular changes observed in fundus examination. In addition, colored fundus photographs (Visucam 500^®^, Zeiss, Germany) and macular thicknesses measured from spectral domain-optical coherence tomography ([SD-OCT]; Spectralis OCT^®^, Heidelberg Engineering, Heidelberg, Germany) obtained during routine examination were recorded from the SD-OCT archive for all patients. After intravitreal anti-VEGF injection, color fundus photographs were obtained only for patients who developed choroidal neovascularization activation or complications/adverse effects. Non-infectious IOI was diagnosed according to the definition on the American Society of Retina Specialists website (https://www.asrs.org/) in the presence of blurred vision and only anterior chamber cells, vitreous cells, or mild fibrous opacity in the vitreous, in the absence of pain, hyperemia, severe vision loss, hypopyon, fibrin reaction, or retinitis focus. These eyes were treated with topical 1% prednisolone acetate (Pred Forte^®^, Allergan Inc, Dublin, Ireland) and follow-up was continued until complete clinical resolution was observed. Based on clinical severity, follow-up examinations were performed every other day for the first week. After the first week, follow-up intervals were extended according to the patient’s clinical presentation.

### Statistical Analysis

All patient data were recorded in the IBM SPSS Statistics (version 24.0, IBM Corp, Armonk, NY, USA) software package and statistical analyses were performed using paired t-test. The results are presented as mean ± standard deviation, and a p-value <0.05 was considered statistically significant.

## Results

Of the 12652 anti-VEGF injections administered for wet AMD, 4796 were aflibercept and 7856 were ranibizumab. Non-infectious IOI was detected in a total of 13 eyes of 13 patients. Therefore, the incidence of non-infectious IOI per injection was 0.1% (13/12,652) and the incidence per patient was 0.66% (13/1,966). Nine (69.2%) of the 13 patients with IOI were male and 4 (30.8%) were female. The patients’ characteristics are summarized in Table 1. The median age was 73 (51-86) years. Nine eyes (69.2%) were pseudophakic. Non-infectious IOI occurred after ranibizumab in 2/7856 eyes (0.02%) and after aflibercept in 11/4796 eyes (0.2%). Median time to presentation to our clinic was 11 (4-21) days. None of the patients had IOI on postoperative day 1. Six patients (46.2%) presented to our clinic with complaints of blurred vision in the first 10 days after the procedure, while in the other 7 patients (53.8%) IOI was detected during routine follow-up examination at 2 weeks. When the patients’ symptoms were examined, we found that 13 patients (100%) had blurred vision, 5 patients (38.5%) had floaters, and 3 patients (23.1%) had photophobia. In wet AMD patients on ongoing anti-VEGF therapy, non-infectious IOI was detected after a median of 7 (2-12) injections.

**Figure 1:**
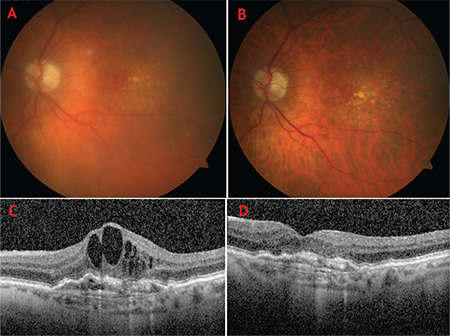
Color fundus and Spectralis optical coherence tomography (OCT) imaging of Patient 1 during intraocular inflammation (A-D). Color fundus image at admission (A) and colored fundus image 1 month after admission (B). Spectralis OCT images before intraocular inflammation (C) and on day 7 of inflammation (D)

According to the patients’ medical records, it was determined that all patients had anterior chamber reaction (Tyndall +1 to +3) and vitritis (grade 1-3) (Figure 1A, B). Hypopyon and fibrin reactions were not observed in any of the eyes (Table 2). The median pre-injection visual acuity of the patients was 0.4 (0.1-0.7; ≤0.1 in 23.1% [3/13]; Snellen chart) and the median visual acuity with IOI was 0.2 (0.05-0.5; ≤0.1 in 30.8% [4/13]). After inflammation resolved, the median visual acuity was 0.5 (0.1-0.7; ≤0.1 in 7.7% [1/13]). We observed that BCVA was significantly reduced during IOI, then increased significantly compared to the inflammation period (p<0.0001). According to the patients’ SD-OCT archives, the median central macular thickness (CMT) before injection was 322 (226-398) µm. The median CMT was 276 (203-370) µm during inflammation and 235 (181-306) µm after the regression of inflammatory findings (Figure 1C, D). Changes in mean CMT due to IOI were not statistically significant compared to pre-injection (p=0.120).

**Figure 2:**
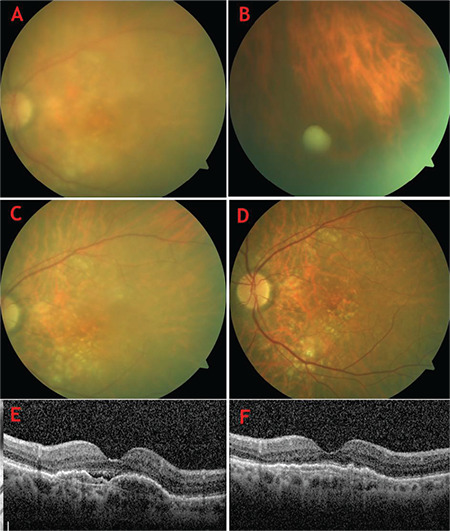
Color fundus and Spectralis optical coherence tomography (OCT) imaging of intraocular inflammation in Patient 2 (A-F). Color fundus photographs at initial presentation (at onset of intraocular inflammation) (A) and at 1 month (B), 2 months (C), and 3 months (D) after presentation. Spectralis OCT images before development of intraocular inflammation (E) and on day 7 of inflammation (F)

In one patient, a vitreous sample had been obtained during follow-up due to grade 2 vitritis and a white opacity in the vitreous (Patient 2, Figure 2A, B, C). Upon detailed review of the patient’s records, we determined that the eye had been treated with intravitreal 1 mg/0.1 mL vancomycin, 2.25 mg/0.1 mL ceftazidime, and 0.4 mg/0.1 mL dexamethasone injections for presumed infectious endophthalmitis. However, no etiological agent was detected in vitreous culture or direct microscopic examination. For all other eyes, topical 1% prednisolone acetate (PredForte^®^) was given every hour for the first 2 days, every 2 hours for the next 3 days, then continued with tapering doses until IOI fully resolved (Figure 2D, E, F).

Patient records indicated that both visual acuity and vitritis improved over time in all patients during follow-up after IOI. The median time to visual acuity recovery was 28 (17-42) days and the median time to vitritis resolution was 32 (20-75) days.

Following functional and anatomical recovery after non-infectious IOI, treatment for wet AMD was continued with the same intravitreal agent in 11 eyes, with a median of 3 (1-12) additional injections. In the other 2 eyes, additional injections were not considered due to scar development. IOI did not recur in any of the eyes and no systemic adverse effects associated with anti-VEGF injection were reported.

## Discussion

Non-infectious IOI can develop after intravitreal injections of ocriplasmin, bevacizumab, ranibizumab, triamcinolone acetonide, and aflibercept.^1,3,4,5,6,7,8,9,10,11,12,13,14^ Its incidence after anti-VEGF injection varies between 0.09% and 0.37% in the literature.^3,4,5^ In the present study, it occurred after intravitreal anti-VEGF injection for wet AMD at a rate of 0.1% of all injections and 0.66% of all patients. The relationship between the indication for anti-VEGF administration and the incidence of IOI is not yet fully understood. In a series of 66 patients who developed IOI after aflibercept injections, Greenberg et al.^1^ reported that the indication for treatment was wet AMD in 74%, macular edema secondary to retinal vein occlusion in 13%, and diabetic macular edema in 10% of the patients.

In our study, the most common symptom at presentation was blurred vision and this symptom was present in all of the patients. The second most common symptom was floaters. The case series reported by Greenberg et al.^1^ is the largest on this subject and the most common symptoms were blurred vision and floaters, consistent with our case series. In our study, the mean time from anti-VEGF administration to presentation due to complaints of blurred vision and floaters was 11.7 days (4-21 days). This is a longer interval compared to previous studies in the literature, in which the mean time to admission was 2.6 to 5 days.^7,8,9,10,11^ We believe the longer time to admission in our patients may be due to the fact that most patients’ inflammation was mild to moderate in severity and therefore they waited for their scheduled follow-up on day 15. One of the patients presented on day 21, which was attributed to low initial visual acuity. However, there were also patients who developed severe inflammation and one-eyed patients who noticed blurred vision early and presented within the first 5 days. In patients with wet AMD who continue anti-VEGF therapy, slit-lamp examinations in addition to follow-up OCT are particularly important for the detection of such inflammation.

Studies have reported the coexistence of anterior chamber reaction and vitritis in 60% to 74% of cases and the presence of severe inflammation (fibrin reaction, presence of hypopyon) in approximately 20%.^1,8,9,10,^^11^ In our study, all patients who developed non-infectious IOI had anterior chamber reaction and vitritis. Only 23% of our patients had +3 Tyndall and/or grade 3 vitritis, while the majority had mild to moderate IOI. None of our patients developed fibrin reaction and/or hypopyon.

In the present study, IOI was associated with a statistically significant decrease in visual acuity. Upon resolution of the inflammation, all patients showed a statistically significant increase in visual acuity. It was reported that patients with IOI may have functional loss in eyes with rapid symptom onset and severe initial inflammation.^8,9,10^ In this case series, as IOI was not severe in any of the eyes, inflammation resolved in 3 to 4 weeks of follow-up and visual acuity returned to the pre-inflammation level.

There are not many studies demonstrating a relationship between non-infectious IOI and activation of wet AMD.^15^ In our study, despite IOI there was a decrease in CMT compared to pre-injection values. However, only SD-OCT findings (subretinal and intraretinal fluid) were evaluated in terms of AMD activation in our case series. Prospective studies with large case series investigating the relationship between IOI development and activation of wet AMD lesions are needed.

Non-infectious IOI can occur regardless of injection number.^5^ Some studies have reported that administration of aflibercept can induce sensitivity and increase the risk of immune reactions with subsequent injections.^10^ Other studies reported that a history of IOI associated with aflibercept injection did not increase the risk or severity of ocular inflammation with subsequent injections.^3,11^ In wet AMD patients who require ongoing treatment, even if IOI occurs it was reported to be unlikely to recur if treatment with the same anti-VEGF agent is continued after the inflammation resolves.^1,7,8,9,10^ Recurrence was not observed in any of the patients in our case series, despite receiving at least 3 anti-VEGF injections during a mean follow-up period of 1 year after IOI.

The mechanism by which non-infectious IOI develops after anti-VEGF injection is not fully understood. Many hypotheses have been proposed regarding the development of inflammation. The most likely hypothesis is that inflammation occurs as a result of an immune reaction caused by the molecular structure of the drug and patient-specific factors. The reported incidence of inflammation is higher after aflibercept than other anti-VEGF agents.^1,3,8,9,10,11,16^ This has been attributed to a proinflammatory interaction between the Fc component in the molecular structure of aflibercept and retinal Fc receptors and/or the fact that aflibercept is a fusion protein with a more viscous structure. We believe that the white opacity seen in the vitreous in one of our patients may be an immune complex formed via a similar mechanism. Other factors that increase the risk of inflammation include contamination of anti-VEGF agents with bacterial endotoxin,^3,4,6^ contamination of the syringes in injection kits with silicon particles,^4^ and inappropriate storage conditions of anti-VEGF agents (cold chain conditions).^3,8^

The most important consideration in cases with non-infectious IOI is the differential diagnosis from infectious endophthalmitis. Several important clinical findings in infectious endophthalmitis facilitate its differential diagnosis. According to the Endophthalmic Vitrectomy Study ^17^, the most important symptoms and findings in infectious endophthalmitis are intense pain, severe vision loss, conjunctival hyperemia, chemosis, fibrin reaction, hypotension, and dense vitreous opacities. The lack of severe visual loss, conjunctival hyperemia, fibrin reaction, hypotension, and dense vitreous opacities in our patients at admission were the main findings for the differential diagnosis of endophthalmitis. In only one of our patients, detection of a whitish opacity in the vitreous (Figure 1) raised suspicion of endophthalmitis and led to collection of a vitreous sample and intravitreal treatment. Examination of the sample revealed no infectious pathogens. During follow-up, the patient’s opacity disappeared within 10 days. The patient was followed up with topical 1% prednisolone acetate until the inflammation resolved.

In patients with non-infectious IOI, systemic and/or topical steroid therapy is recommended depending on the severity of the disease. In most cases, topical steroid therapy is sufficient. All patients in our series were treated with topical steroid therapy and their inflammation resolved. Systemic steroid therapy was not needed in any of our cases. The median time to functional recovery was 28 days and the median time to inflammation resolution was 32 days. No local or systemic adverse effects of topical steroid therapy were observed.

### Study Limitations

The most important limitations of our study are that it was retrospective and included only wet AMD patients. Another limiting factor was the small number of cases.

## Conclusion

Non-infectious IOI following anti-VEGF therapy is a rare complication. The clinical course is mild in many patients. However, it is important to carefully differentiate the condition from infectious endophthalmitis. Patients should be followed very closely and those showing clinical deterioration should be presumed to have infectious endophthalmitis and treated accordingly. Non-infectious IOI responds well to topical steroid therapy. Functional recovery is usually seen in 3 to 4 weeks. After inflammation has completely resolved, treatment with the same anti-VEGF agent can be continued in patients with AMD reactivation.

## Figures and Tables

**Table 1:**
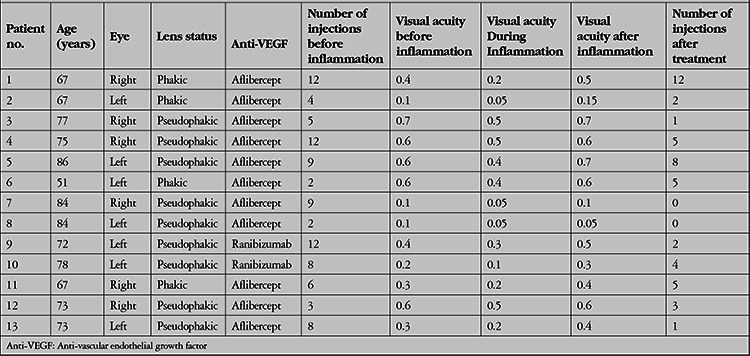
Demographic characteristics and clinical findings of the patients

**Table 2:**
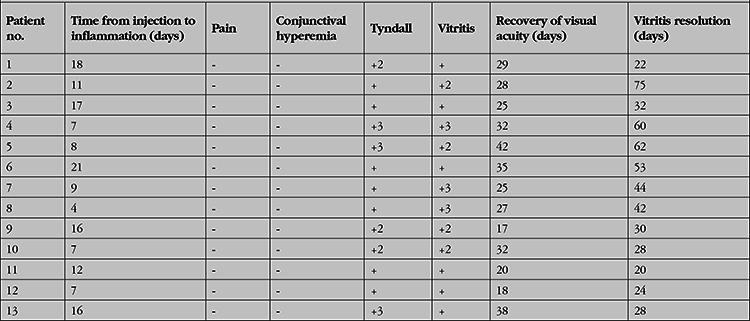
Features and recovery times of the patients’ intraocular inflammation
